# Regulatory mechanisms of glucose absorption in the mouse proximal small intestine during fasting and feeding

**DOI:** 10.1038/s41598-023-38024-w

**Published:** 2023-07-05

**Authors:** Chisato Nakamura, Noriko Ishizuka, Kanako Yokoyama, Yuyu Yazaki, Fumiya Tatsumi, Naotaka Ikumi, Wendy Hempstock, Akira Ikari, Yuta Yoshino, Hisayoshi Hayashi

**Affiliations:** 1 Laboratory of Physiology, Graduate School of Nutritional and Environmental Sciences, University of Shizuoka, 52-1 Yada, Suruga-ku, Shizuoka, 422-8526 Japan; 2 Department of Nursing, School of Nursing, University of Shizuoka, 52-1 Yada, Suruga-ku, Shizuoka, 422-8526 Japan; 3 Laboratory of Biochemistry, Gifu Pharmaceutical University, Gifu, 501-1196 Japan

**Keywords:** Physiology, Gastroenterology

## Abstract

Fasting is known to alter the function of various organs and the mechanisms of glucose metabolism, which affect health outcomes and slow aging. However, it remains unclear how fasting and feeding affects glucose absorption function in the small intestine. We studied the effects of the fasting and feeding on glucose-induced short-circuit current (*I*_*sc*_) in vitro using an Ussing chamber technique. Glucose-induced *I*_*sc*_ by SGLT1 was observed in the ileum, but little or no *I*_*sc*_ was observed in the jejunum in ad libitum-fed mice. However, in mice fasted for 24–48 h, in addition to the ileum, robust glucose-induced *I*_*sc*_ was observed over time in the jejunum. The expression of SGLT1 in the brush border membranes was significantly decreased in the jejunum under fed conditions compared to 48 h fasting, as analyzed by western blotting. Additionally, when mice were fed a 60% high glucose diet for 3 days, the increase in glucose-induced *I*_*sc*_ was observed only in the ileum, and totally suppressed in the jejunum. An increase in Na^+^ permeability between epithelial cells was concomitantly observed in the jejunum of fasted mice. Transepithelial glucose flux was assessed using a non-metabolizable glucose analog, ^14^C-methyl α-d-glucopyranoside glucose (MGP). Regardless of whether fed or fasted, no glucose diffusion mechanism was observed. Fasting increased the SGLT1-mediated MGP flux in the jejunum. In conclusion, segment-dependent up- and down-regulation mechanisms during fasting and feeding are important for efficient glucose absorption once the fast is broken. Additionally, these mechanisms may play a crucial role in the small intestine's ability to autoregulate glucose absorption, preventing acute hyperglycemia when large amounts of glucose are ingested.

## Introduction

It is well known that caloric restriction has beneficial effects on aging in many animal species. Recent studies have shown that the benefits of fasting are not simply the result of reducing body weight or free radical production. Fasting elicits evolutionally adaptive responses that are integrated between organs and improves glucose regulation, resulting in effects on health outcomes and slowing of the aging disease process^1^. Overnight fasting of mice is a common procedure performed for evaluation, especially of blood glucose, in association with metabolic disease^2^. This makes it a confounding factor when trying to properly assess the mechanism of small intestinal glucose absorption.

However, it remains unclear how small intestine glucose absorption responds during fasting and feeding. The mechanism by which ingested glucose is absorbed into the epithelial membrane of the small intestine against a concentration gradient was first proposed by Crane in 1960^3^. The driving force for the glucose transporter is provided by the inward Na^+^ concentration gradient maintained by the Na^+^ pump in the basolateral membrane. Wright et al*.* showed that the molecular identity of the Na^+^-dependent glucose transporter is sodium-dependent glucose transporter (SGLT) 1^4^. As for the molecular identity of basolateral glucose transporter, the facilitated glucose transporter (GLUT2) found expressed in the liver is also exclusively expressed in the basolateral membrane of enterocytes, indicating that it is a glucose diffusion exit pathway^5^. This mechanism of SGLT1 and GLUT2-mediated glucose absorption in enterocytes is widely accepted^6^.

In addition to the transporter-mediated glucose absorption mechanism (saturable component), it was also shown that there is a co-existing diffusion mechanism (un-saturable component) of glucose when rat intestine is perfused with high concentrations (~ 100 mM) of glucose^7^. In regards to un-saturable pathways, Pappenheimer et al*.* proposed a hypothesis that intercellular spaces in the epithelial cells open and glucose diffuses through this pathway, which is based on a series of studies using morphological changes observed by electron microscopy as well as impedance measurements^8–10^. Kellett et al*.* proposed an alternative absorption mechanism for the diffusion pathway of glucose. GLUT2, a diffusion transporter that normally functions as a glucose efflux mechanism in the basolateral membrane, would be inserted into the luminal membrane when high concentrations of glucose are present in the luminal perfusate in vivo^11,12^. Kellett's idea has not been widely accepted because the mechanism of glucose absorption in the small intestine is not impaired in Fanconi-Bickel syndrome, a deficiency of GLUT2 in humans, or in GLUT2-deficient mice^13–15^. Recently, Wright et al*.* used positron emission tomography (PET) and a glucose tracer to investigate the mechanism of glucose absorption in SGLT1-deficient and GLUT2-deficient mice^16^. Although SGLT1 is required for the rapid glucose absorption mechanism in the small intestine, they also observed that glucose is absorbed slowly in SGLT1-deficient mice and that glucose absorption function is not impaired in GLUT2-deficient mice. Thus, the existence and significance of the diffusional transport component in glucose transport is still being debated, and even now the results of previous studies cannot be fully explained.

Furthermore, we have demonstrated that the Na^+^ which is absorbed by Na^+^-glucose cotransport is recycled back into the lumen via paracellular Na^+^ conductance through claudin-15, which is driven by Na^+^ cotransport-induced luminal potential^17^. However, little research has been done on how fasting and feeding affects the ion permeability of the small intestinal tight junctions. We therefore studied the effects of fasting and feeding on glucose-induced short-circuit current (*I*_*sc*_) and measured unidirectional glucose fluxes in vitro using an Ussing chamber technique.

## Material and methods

### Animals

Male mice (C57BL/6JJcl, 6–19 weeks) were purchased from Clea Japan (Tokyo, Japan). Mice were fed a standard pellet diet (MF, Oriental Yeast, Tokyo, Japan) and water was provided ad libitum until use in experiments. Animals were bred in the University of Shizuoka animal facility with a room temperature of 23 ± 1 °C, humidity of 55 ± 5%, and lighting time of 12 h (8:00–20:00). In experiments examining the effect of fasting on glucose transport function, animals were fed a normal diet (MF) as a control group. The effects of fasting on glucose transport function were examined in 24-h fasting and 48-h fasting groups without restricting water. Prior to the Ussing chamber experiments, mice were housed individually, fasted, and allowed to drink water ad libitum. In consideration of the circadian rhythm, functional measurements were performed between 1:00 p.m. and 5:00 p.m. The sample size was calculated using the Power and Sample Size Calculation program (version 3.12, Vanderbilt University, Nashville, Tennessee, USA), based on measuring SGLT1 activity through short-circuit current change. To detect a difference of 50 μA/cm^2^ in the mean short-circuit current between the two groups, we estimated the sample size with a significance level of α = 0.05, power = 0.8, and a standard deviation of 30 for the baseline short-circuit current, requiring seven mice in each group. Mice under different conditions, including ad libitum, 24-h fasting, and 48-h fasting conditions, were used to measure the baseline electrical parameters, SGLT1 activity, and CFTR activity. No mice died during the experiment. In total, 15 mice were used under ad libitum conditions, and 12 mice each under 24-h fasting and 48-h fasting conditions for the measurements. However, some data were missing due to electrode trouble, which made it impossible to measure in specific chambers. All animal experimental procedures and handling were approved by the Animal Care and Use Committee of the University of Shizuoka (reference nos. 185196 and 225345) and conducted in accordance with the Guidelines and Regulations for the Care and Use of Experimental Animals by the University of Shizuoka.

### Tissue preparation

**Figure 1:**
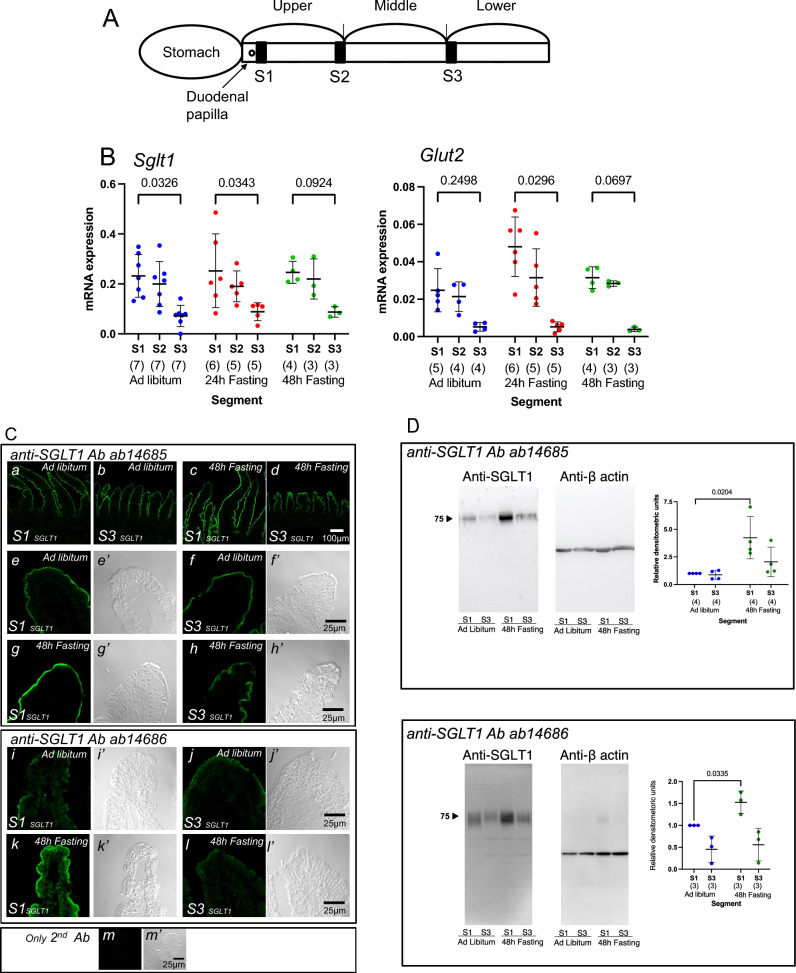
Effect of fasting on expression of sodium-dependent glucose transporter 1 (SGLT1) and facilitated glucose transporters (GLUT2) along the intestinal axis. Designation of the segments of the small intestine (**A**). The small intestine was divided into three segments. The distal part of the duodenal papilla is designated as S1 segment, the most distal part of upper segment is designated as S2 segment, and the proximal part of the third part is designated as S3 segment. The mRNA expression levels and effect of fasting on *Sglt1* and *Glut2* (**B**). Data are presented as mean ± standard deviation (SD). Two-way ANOVA followed by Tukey’s multiple comparisons test. *P* values are shown with numbers. Numbers in parentheses indicate the number of animals. To determine the specificity of immunofluorescent staining (**C**) and Western blotting (**D**) for SGLT1, two antibodies (ab14685 and ab14686) were used. The upper panel shows images with ab14685 antibody (**C**). The top row images (a–d) show a low magnification view (20× objective). The middle and bottom two rows (e–h) display higher magnification magnified images (63× objective) with their respective differential interference contrast (DIC) images, indicated by lowercase letters with a prime mark, to demonstrate the specificity of the signal. The middle panel shows images with ab14686 antibody. The top row images (i and j) show in ad libitum mice and bottom row (k and l) shows in 48-h fasted mice. The bottom panel (m) depicts the negative control using only secondary antibodies. Images are representative of three animals. To compare fluorescence levels, the images were taken under the same configurations in each condition. Brush border membranes vesicles (BBMVs) were prepared from intestinal epithelial cells and analyzed by western blotting (**D**). The image illustrated is representative of four and three similar experiments with ab14685 and ab14686, respectively. The graph shown is a summary of the quantitation of the SGLT1 signals. Numbers in parentheses indicate the number of animals. Relative values are shown with S1 from ad libitum-fed mice as 1. Each lane was loaded with 5 μg of total protein, the membrane stained with β-actin is shown as a loading control.

Mice were anesthetized by intraperitoneal administration of a mixture of three anesthetic drugs: 30 µg/mL of medetomidine (Nippon Zenyaku Kogyo, Fukushima, Japan), 0.4 mg/mL of midazolam (Teva Pharma Ltd., Nagoya, Japan) and 0.5 mg/mL of butorphanol (Meiji Seika, Tokyo, Japan) at 10 µL/g body weight. After anesthesia, the whole small intestine was excised and divided into three segments in length (Fig. 1A). The distal part of the duodenal papilla was designated as S1 segment, the most distal part of upper segment was designated as S2 segment, and the proximal part of the third part was designated as S3 segment (Fig. 1A). The intestine was incised along the longitudinal axis, and the contents were removed by washing with ice-cold standard Ringer’s solution gassed with 95% O_2_/5% CO_2_. Specimens for Ussing chambers were prepared as follows: the intestine was fixed with small needles, mucosal side down, on a silicone-rubber-covered Petri dish filled with ice-cold standard Ringer’s solution and the muscle layer was removed using fine forceps under a stereomicroscope.

### Measurement of electrical parameters in Ussing chambers

The measurement of electrical parameters in Ussing chambers was performed as previously described^18^. The mucosa-submucosa specimens were mounted in a pair of Ussing chambers with circular windows of 5 mm in diameter. The mucosal and serosal sides were each filled with 5 mL of Ringer’s solution and maintained at 37 °C by perfusion from an external thermostatic bath. The normal Ringer’s solution (containing in mM: 119 NaCl, 21 NaHCO_3_, 2.4 K_2_HPO_4_, 0.6 KH_2_PO_4_, 1.2 CaCl_2_, 1.2 MgCl_2_, 0.5 L-glutamine, and 10 µM indomethacin) was gassed with 95% O_2_/5% CO_2_ (pH 7.4). Calomel electrodes connected with 1 M KCl/2% agar salt bridges were placed on both sides of the chamber for potential difference measurement, and Ag/AgCl electrodes connected with 1 M NaCl/2% agar salt bridges were placed for current flow. The short-circuit current (*I*_*sc*_) was measured under short-circuit conditions by connecting these electrodes to a short-circuit current measurement system (Nihon Kohden CEZ-9100, Tokyo, Japan). The transepithelial conductance (*G*_*t*_) was determined by Ohm's law from the change in current generated by applying external voltage pulses of ± 5 mV every 5 min, and *I*_*sc*_ and *G*_*t*_ were recorded by a chart recorder (DKK-TOA corporation, Japan). The dilution potential was measured under open-circuit conditions, and the transepithelial potential was determined by Ohm's law from the voltage change measured with an external current load of ± 20 µA every 5 min. To measure the dilution potential, the buffer on one side (either Mucosal or Serosal side) was replaced with the above normal Ringer’s solution containing 60 mM NaCl and 120 mM mannitol as previously described^17,19^.

### Measurement of methyl α-d-glucopyranoside flux

Measurement of Methyl α-d-glucopyranoside (a nonmetabolizable analog of glucose, MGP) flux in Ussing chambers was performed as previously described^17^. After mounting the preparations in Ussing chambers, the mucosal or serosal side was replaced with a 30 mM MGP containing solution (containing in mM: 30 MGP, 104 NaCl, 21 NaHCO_3_, 2.4 K_2_HPO_4_, 0.6 KH_2_PO_4_, 1.2 CaCl_2_, 1.2 MgCl_2_, 0.5 L-glutamine, and 10 µM indomethacin). And the opposite side, which did not contain MGP, was iso-osmotically replaced with Mannitol. The solution containing MGP was labeled with ^14^C-MGP (12.5 kBq/5 mL). After 20 min, 0.5 mL of the Ringer’s solution was collected from the cold side and replaced with the same volume of fresh solution every 20 min. The specific activity of ^14^C-MGP was calculated by collecting 20 µL from the label side at the beginning and at the end of the measurement and used for unidirectional flux calculation. The collected samples were mixed with 5 mL of liquid scintillation cocktail (Ultima Gold, Perkin Elmer Norwalk, Connecticut) and the β-rays were measured in a liquid scintillation counter (LSC-8000, Hitachi-Aloka Medical, Tokyo, Japan).

### Quantitative real-time RT-PCR

The excised intestines were cut open along the longitudinal axis and the luminal contents were washed with standard Ringer’s solution. The tissue was then immersed in 0.5 ml RNA later solution (Ambion Inc., Austin, TX). The specimens were fixed with RNA later solution and stripped of muscle layer under a stereomicroscope. Total RNA extraction was performed using the NucleoSpin RNA extraction kit (Macherey-Nagel, Germany) according to the manufacturers’ instructions. cDNA synthesis was performed using Prime Script RT Master mix (Takara, Shiga, Japan). The primers used for real-time PCR are listed in Table 1, and beta-actin was used as the housekeeping gene. The Thermal Cycler Dice TP700 (Takara, Shiga, Japan) was used for PCR experiments, and the relative change of gene expression was analyzed by the ∆∆ Ct method^20^.

**Table 1:** List of primers used for quantitative real-time PCR.

Gene name (Alias)	Primer	Sequence
Actb(β-actin)	Forward	CATCCGTAAAGACCTCTATGCCAAC
	Reverse	ATGGAGCCACCGATCCACA
Slc5a1(SGLT1)	Forward	TCTTCACCATGGACATCTACACCAA
	Reverse	GATGCCAATCAGCACGAGGA
Slc2a2(GLUT2)	Forward	GGCATCAGCCAGCCTTGTGTA
	Reverse	CATGCCAATCATCCCGGTTAG
Cldn2(claudin-2)	Forward	AATTCCCGTACATTTGTGGGTCA
	Reverse	ACCTTGGCTTCTGGGCAATTC
Cldn7(claudin-7)	Forward	CAGAGCACCGGCATGATGA
	Reverse	TGGCGACAAACATGGCTAAGAA
Cldn15(claudin-15)	Forward	CAACGTGGGCAACATGGA
	Reverse	TGACGGCGTACCACGAGATAG

### Immunofluorescence staining

Immunofluorescent staining of claudin-2, -7, and -15 was performed using the same method as previously published^17,21^. For SGLT1, different staining methods were used. Mice were anesthetized intraperitoneally with 10 µL/g body weight of a mixture of three anesthetics (same as above). The small intestine was excised and opened longitudinally. Each segment was then rinsed with ice cold PBS and embedded in Tissue-Tek O.C.T. compound (Sakura Finetek, Tokyo, Japan). The samples were frozen at − 80 °C until sectioning. The specimens were sectioned to 7 µm thickness using a cryostat (Cryostar NX50 HD, PMC, Tokyo, Japan) and put on circular coverslips (13 mm in diameter, Matsunami Glass, Osaka, Japan). The coverslips were incubated in 95% ethanol on ice for 30 min followed by incubation in acetone for 1 min. For antigen retrieval, we applied autoclave heating methods as published previously^25^. The coverslips were immersed in EDTA-HEPES buffer (2 mM EDTA, 200 mM HEPES, pH 8.0 for ab14685, 2 mM EDTA, 200 mM Tris, pH 9.0 for ab14686) and autoclaved at 121 °C for 20 min. After the antigen retrieval treatment, the coverslips were washed three times in PBS for 5 min. The sections were pre-blocked with 5% skim milk and 0.1% triton in PBS for 30 min. The sections were next stained with rabbit anti-SGLT1 antibody (ab14685, 1:200 dilution: Abcam, Cambridge, UK, Antibodies are directed against a synthetic peptide corresponding to human SGLT1, from amino acids 600 to 700; ab14686, 1:200 dilution: Abcam, Antibodies are directed against a synthetic peptide corresponding to human SGLT1, from amino acids 402 to 422) for 30 min. After washing 3 times with PBS, the sections were stained with Alexa 488-conjugated goat anti-rabbit antibody (1:1000 dilution, Abcam, Cambridge, UK) for 30 min. The coverslips were mounted on slides with Fluoromount-G (Southern Biotechnology Associates, Birmingham, AL). SGLT1 staining was visualized through a laser scanning microscope (LSM700; Zeiss, Oberkochen, Germany).

### Preparation of brush border membrane vesicles and western blotting

Brush border membrane vesicles (BBMV) were prepared using a Mg precipitation method^22^ with modifications. Approximately 3 cm was taken from each segment of the small intestine, and everted small intestinal sacs were prepared. The everted sacs were then placed in a 50 mL tube containing 30 mL of Ca^2+^ chelating buffer containing 5 mM EDTA and 5 mM EGTA then incubated on ice for 60 min. After incubation, the everted sacs were shaken vigorously, and the epithelial cells were isolated by centrifugation for 5 min at 200 g. The resulting pellet was resuspended in ice-cold medium containing 300 mM mannitol, protease inhibitor (Complete, Roche, Branchburg, USA), and 10 mM HEPES/Tris (pH 7.4) and homogenized with a sonicator 10 times for 1 s each time. MgCl_2_ was added to a final concentration of 10 mM, followed by incubation for 30 min on ice and centrifugation for 15 min at 2300 g. The supernatant was collected and subjected to centrifugation for 90 min at 18,500 g. The resulting pellet containing the brush-border vesicles was resuspended in 2× concentrated Laemmli sample buffer. The samples were subjected to SDS-PAGE and transferred onto a nitrocellulose membrane. Blots were blocked with 5% milk and 0.1% Triton in PBS and exposed to the primary antibody (1:1000 dilution, anti-SGLT1, Abcam, ab14685 and ab14686). Horseradish peroxidase-conjugated secondary antibody (1:10,000 dilution, Donkey Anti-Rabbit IgG, Jackson Immuno Research, 711-035-152) was then used, and the immunoreactive bands were visualized with Super Signal West Dura Extended Duration substrate (Thermo Fisher Scientific) using a Fusion Solo (Vilber, Lourmat, France). β-actin (1:1000 dilution, Abcam, ab75186) was used as a loading control.

### Statistics

Data are presented as mean ± standard deviation (SD). Two-tailed Student’s *t*-test was used to compare the data of two groups. One-way or Two-way of Analysis of variance (ANOVA) was used for the data with three or more groups, and Tukey's multiple comparison test was used for the post hoc test (GraphPad Prism 9, GraphPad Software, La Jolla, CA). The *K*_*m*_ and *V*_*max*_ values for the glucose induced-*I*_*sc*_ response were determined by fitting the curve to the Michaelis–Menten equation using nonlinear regression using GraphPad Prism software. *P* values < 0.05 were considered to be statistically significant. In the figures, n is indicated by a number in parentheses or stated in the legend.

## Results

### Effect of fasting on mRNA abundance of glucose transporters along the intestinal axis

Segmental differences in gene expression along the length of the small intestine of sodium-dependent glucose transporter 1 (SGLT1) and facilitated glucose transporters (GLUT2) in the mouse have been studied in detail^23^. It was reported that the expression levels of SGLT1 protein is the highest in the jejunum, followed by the duodenum, and finally the ileum^24^. We first examined the mRNA abundance of *Sglt1* and *Glut2* transporters in the S1-S3 segments of ad libitum-fed mice (blue symbols). As shown in Fig. 1B, *Sglt1* and *Glut2* mRNA are expressed at higher levels in S1 segments compared to S3 segments, consistent with a previous study^23^.

We next examined the effect of 24-h fasting (Fig. 1B, red symbols) on mRNA abundance. Segment-specific mRNA expression patterns were not significantly altered by 24-h fasting conditions. In addition, the fasting time was extended and the effect of 48-h fasting on mRNA expression (green symbols) was also examined. In the 48-h fasting conditions, no obvious changes were observed in either *Sglt1* or *Glut2* transporters compared to ad libitum fed mice. We next examined the effect of fasting on expression of SGLT1 protein in the S1 segment by immunofluorescence (Fig. 1C, a–d). To determine the specificity of immunofluorescent staining (Fig. 1C) and Western blotting (Fig. 1D) for SGLT1, we used two antibodies (ab14685 and ab14686) commercially available from Abcam. It has been shown that ab14686 antibody has clearly distinguishable SGLT1-specific signals, which were not visualized in the small intestine of SGLT1-deficient mice^24^. In ad libitum fed mice, SGLT1 signals localized in the epithelial cells in a low magnification view (e–h and i–l, ab14685 and ab14686, respectively), consistent with a previous study^24^. To assess the subcellular distribution of SGLT1 in enterocytes, higher magnification images and differential interference contrast (DIC) images (Fig. 1C, e′–m′, indicated by lowercase letters with a prime symbol) were obtained at the same time. In the S1 and S3 segments under 48-h fasting conditions, no obvious changes were observed in the subcellular localization of SGLT1, which was exclusively expressed in the microvilli, compared to that in fed conditions (Fig. 1C, e and f, i and j, ab14685 and ab14686, respectively). However, in the fasted S1 segment (g and k, ab14685 and ab14686, respectively), the fluorescent signal of SGLT1 was stronger than that in the fed conditions. This could be due to an increase in SGLT1 expression just below the plasma membrane. Therefore, brush border membranes vesicles (BBMVs) were prepared from intestinal epithelial cells and analyzed by western blotting (Fig. 1D). Recently, it has been reported that the mouse heart expresses a non-functional truncated form of SGLT1 (Exon 9–14, 978–1998 bp, 292 amino acids, predicted M.W. 33 kDa)^25^. The open reading frame (ORF) of mouse *Sglt1* (NM_019810.4) mRNA is 1998 bp. We used RT-PCR primers for *Sglt1* (Table 1), which can also amplify the splice variant of mRNA, 1211–1309 (98 bp). Therefore, it is possible that the truncated form of SGLT1 is expressed in the small intestine. Since electrogenic glucose absorption activity (Fig. 3) is ultimately dependent on the amount of SGLT1 protein in the apical membrane, we quantified SGLT1 protein expressed on the apical membrane by isolating the BBMVs. As shown in Fig. 1D (upper and lower, ab14685 and ab14686, respectively), 80 kDa SGLT1 in the brush border membrane was significantly increased during fasting and no small molecule splice variant of SGLT1 was observed. These results suggest that SGLT1 (80 kDa) expression is upregulated in the brush border membrane at the S1 site during fasting and downregulated during feeding.

### Effect of fasting on basal electrophysiological parameters along the intestinal axis

**Figure 2:**
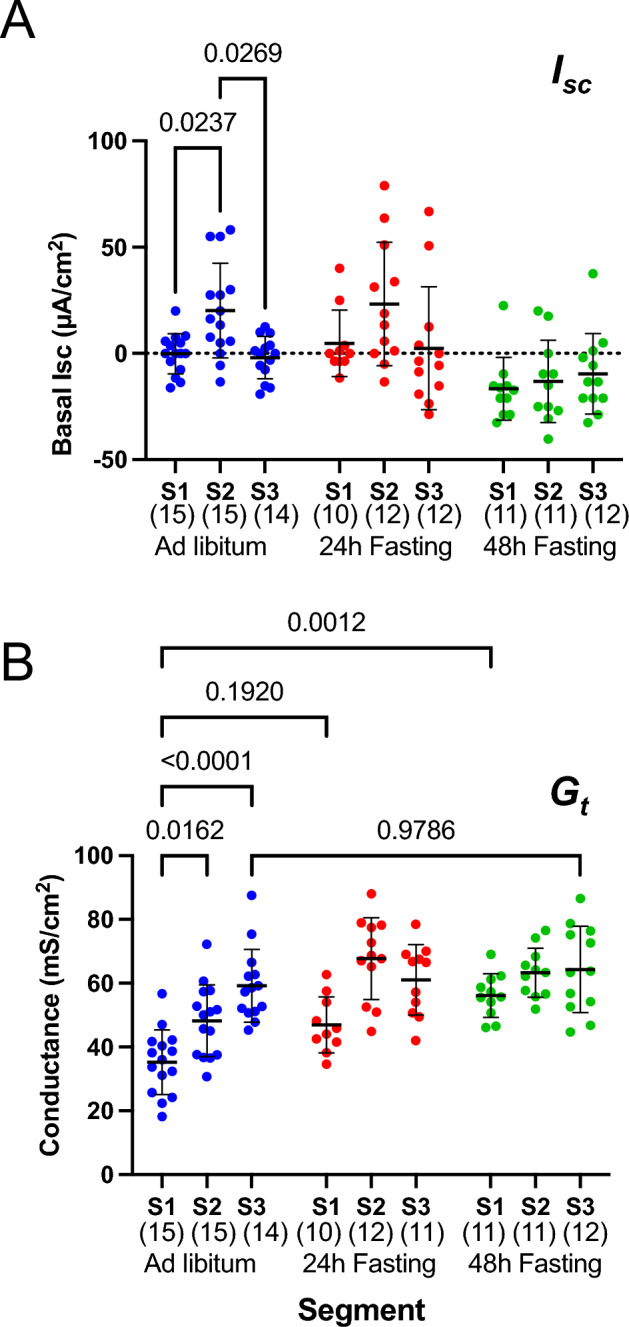
Effect of fasting on basal short-circuit current (*I*_*sc*_) and transepithelial conductance (*G*_*t*_) along the intestinal axis. Basal *I*_*sc*_ (**A**) and *G*_*t*_ (**B**) measured under short-circuit conditions. Designation of segment is as shown in Fig. 1A. Data are presented as mean ± SD. Numbers in parentheses indicate the number of animals. *P* values are shown with numbers, Two-way ANOVA.

We first examined the effect of fasting on the electrophysiological parameters in the small intestine using the Ussing chamber technique. In ad libitum-fed mice (Fig. 2A, blue symbols), a slight short-circuit current (*I*_*sc*_) was observed in the baseline condition, but no significant changes were observed at any segment in the 24- (red symbols) and 48-h fasted (green symbols) mice (Fig. 2A), compared to that ad libitum-fed mice. The effect of fasting on transepithelial conductance (*G*_*t*_), which is mainly reflected by ion permeability of the tight junctions in the small intestine^17^, was also examined (Fig. 2B). In ad libitum-fed mice (blue symbols), *G*_*t*_ was gradually increased from the proximal to the distal segment and the values were significantly higher in the S2 and S3 segments than that in the S1 segment. In the S1 segment, *G*_*t*_ increased with prolonged fasting time and significantly increased after 48 h of fasting compared to ad libitum-fed mice. However, in the S3 segment, fasting-induced* G*_*t*_ changes were not observed.

### Effect of fasting on glucose-induced short-circuit current (*I*_*sc*_) along the intestinal axis

**Figure 3:**
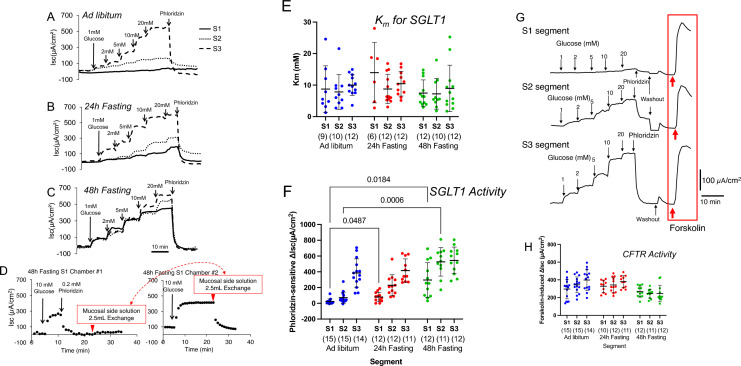
Effect of fasting on glucose-induced short-circuit current (Δ*I*_*sc*_) along the intestinal axis. A representative trace in each segment under ad libitum and fasting conditions is shown. Ad libitum fed mice (**A**), 24-h fasted mice (**B**) and 48-h fasted mice (**C**). The final concentration of glucose is shown in mM. 100 µM phloridzin was added to mucosal side. The effect of fasting on phloridzin degradation is shown (**D**). To examine whether phloridzin degrades in the chamber, 0.2 mM phloridzin was added to one chamber after the addition of glucose and allowed to incubate for 10 min. As indicated by the red arrowhead, half the volume (2.5 mL) of the mucosal solution from the first chamber (containing the phloridzin) was added to the other chamber to which no phloridzin had been added. A representative trace from three experiments is shown. The summary of the Michaelis–Menten constant (*K*_*m*_) for SGLT1 (**E**). The summary of phloridzin-sensitive Δ*I*_*sc*_ (**F**). *P* values are shown with numbers, Two-way ANOVA. Representative traces of CFTR activity measurements are shown (**G**). After measuring SGLT1 activity, the mucosal and serosal sides of the Ussing chamber were washed with fresh Ringer's solution. After stabilizing the baseline short-circuit current, 10 µM forskolin, an inhibitor of adenylate cyclase, was added to serosal side of the chambers. The summary of forskolin-induced Δ*I*_*sc*_ (**H**). Data are presented as mean ± SD. Numbers in parentheses indicate the number of animals.

We assessed the function of SGLT1 in each intestinal segment using glucose-induced short circuit current (Δ*I*_*sc*_) as an indicator. We first measured glucose-induced Δ*I*_*sc*_ in ad libitum-fed mice. Upon the addition of glucose to the mucosal side of the Ussing chamber, glucose-induced Δ*I*_*sc*_ was increased in a dose-dependent manner in the S3 segment (Fig. 3A, dashed line). Although, the expression level of *Sglt1* mRNA is higher in S1 segment (Fig. 1B), glucose-induced Δ*I*_*sc*_ was only slight (Fig. 3A, solid line). Glucose-induced Δ*I*_*sc*_ was not observed in 6 out of 15 mice in the S1 segment and 1 out of 15 mice, in the S2 segment. In contrast, in the S3 segment, glucose-induced Δ*I*_*sc*_ was observed in all 14 mice. This change in *I*_*sc*_ conformed to Michaelis–Menten kinetics. The Michaelis–Menten constant (*K*_*m*_) was not significantly different under any of the conditions (Fig. 3E), suggesting that the properties of SGLT1 transport were not changed under fasting conditions.

To investigate whether glucose-induced Δ*I*_*sc*_ is due to SGLT1 or not, a specific inhibitor of SGLT1, phloridzin^26^, was added to luminal side after addition of 20 mM glucose (Fig. 3A–C). Glucose-induced Δ*I*_*sc*_ was almost completely suppressed in all segments after the addition of phloridzin, suggesting that the glucose-induced Δ*I*_*sc*_ increment was via SGLT1. Under ad libitum-fed conditions, phloridzin-sensitive Δ*I*_*sc*_ was lower at the S1and S2 segments compared to the S3 segment (Fig. 3F, blue symbols).

To examine whether phloridzin degrades in the chamber, 0.2 mM phloridzin was added to one chamber after the addition of glucose and allowed to incubate for 10 min. As indicated by the red arrowhead (Fig. 3D), half the volume (2.5 mL) of the mucosal solution from the first chamber (containing the phloridzin) was added to the other chamber to which no phloridzin had been added. A representative trace from three experiments is shown. As shown in Fig. 3D, the glucose-induced short-circuit current was totally suppressed in the second chamber, even though the concentration of phlorizin had been diluted to 0.1 mM. This indicates that under in vitro conditions, phloridzin sensitivity does not change, and phloridzin, if altered, was likely rapidly metabolized and inactivated. However, under in vivo conditions, the potential change in phlorizin sensitivity needs to be examined in the future.

There was a discordance between the abundance of mRNA (Fig. 1B) and phloridzin-sensitive Δ*I*_*sc*_ (Fig. 3A, F) along the intestinal axis. A possible reason for this is that the tissue is damaged. This possibility was assessed by electrogenic Cl^−^ secretion, which is mainly mediated by cystic fibrosis transmembrane conductance regulator (CFTR). Representative traces of CFTR activity measurements are shown in Fig. 3G. After measuring SGLT1 activity, the mucosal and serosal sides of the Ussing chamber were washed with fresh Ringer's solution. After stabilizing the baseline *I*_*sc*_, forskolin, an inhibitor of adenylate cyclase, was added to serosal side of the chambers. The increase in *I*_*sc*_ was used to measure CFTR activity. This forskolin-induced increase in *I*_*sc*_ was also observed in the S1 segment, where glucose-induced *I*_*sc*_ was not observed, as well as in the 48-h fasting group. In all segments, forskolin-induced Δ*I*_*sc*_ was 300–400 µA/cm^2^ in ad libitum-fed mice (Fig. 3H, blue symbols), suggesting that tissue integrity was maintained even in the S1 segment under fasting conditions.

We next examined the effect of 24-h fasting on glucose-induced Δ*I*_*sc*_ (Fig. 3F, red symbols). In the S1 segment, glucose-induced Δ*I*_*sc*_ increased significantly compared to ad libitum-fed mice. Although it did not reach significance levels, obvious glucose-induced Δ*I*_*sc*_ was observed in S2 segments compared to ad libitum-fed mice (Fig. 3F, red symbols). Forskolin-induced Δ*I*_*sc*_ was approximately 400 µA/cm^2^ after 24 h of fasting, and no significant difference was observed in any of the segment compared to the ad libitum-fed mice (Fig. 3H, red symbols).

Next, we examined the effect of prolongation of fasting on the glucose-induced Δ*I*_*sc*_. As shown in Fig. 3C, when mice were fasted for 48 h, robust glucose-induced Δ*I*_*sc*_ was observed in the S1 segment (solid line). The phloridzin-sensitive Δ*I*_*sc*_ was also increased compared to that in the ad libitum-fed mice in the S1 and S2 segment (Fig. 3F, green symbols), but not the S3 segment. However, forskolin-induced Δ*I*_*sc*_ was slightly decreased compared to that in the 24-h fasted mice in all segments (Fig. 3H, green symbols).

### Effect of a high glucose diet on glucose-induced short-circuit current (*I*_*sc*_)

The functional activity of nutrient transporters in the small intestine is regulated by several different regulatory patterns in response to the intake amount and the nutritional status^27^. For nutrients that are metabolized for energy, such as sugars and amino acids, there is a positive relationship between the nutrient intake and transporter activity^27^. Indeed, it has been reported that SGLT1 mRNA and protein levels are increased when rats are fed with a high glucose diet^28^. However, this mechanism is inconsistent with the fact that in natural conditions, one cannot predict when the next intake will occur or how much will be consumed. Therefore, we evaluated the glucose transport capacity of the upper small intestine after high glucose intake, fasting, and refeeding.

**Figure 4:**
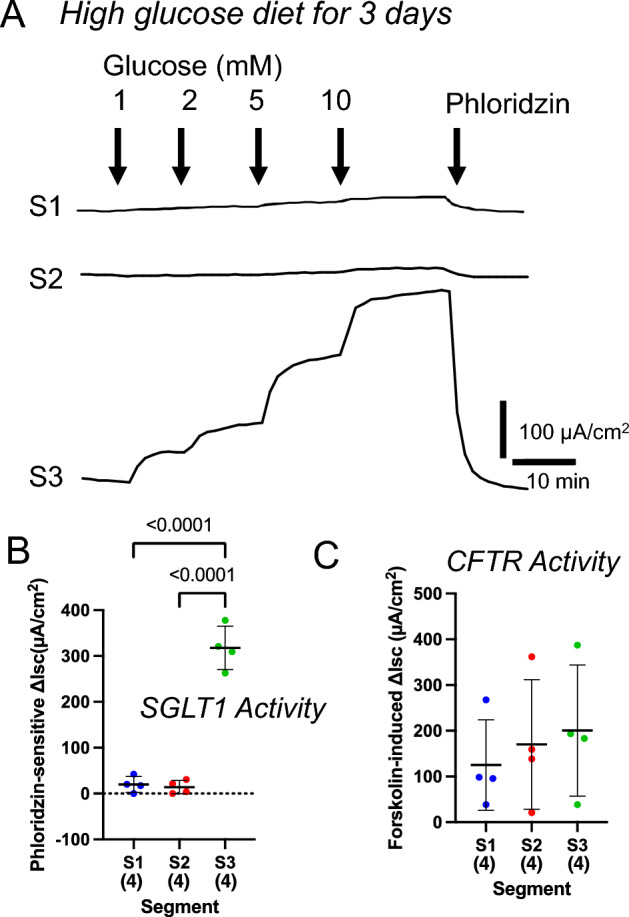
Effect of a high glucose diet on glucose-induced short-circuit current (Δ*I*_*sc*_). Representative traces are shown from four experiments (**A**). Mice were fed with a high glucose (60%) diet for 3 days. The summary of phloridzin-sensitive Δ*I*_*sc*_ (**B**) and forskolin-induced Δ*I*_*sc*_ (**C**). Data are presented as mean ± SD. Numbers in parentheses indicate the number of animals. *P* values are shown with numbers, One-way ANOVA.

When mice were fed with a 60% high glucose diet for 3 days, no increase in phloridzin-sensitive Δ*I*_*sc*_ was observed in the S1 and S2 segments (Fig. 4A, B). In contrast, a small phloridzin-sensitive Δ*I*_*sc*_ was observed at the S2 segment in the ad libitum-fed mice on a normal diet (Fig. 3F) compared to the 60% high glucose diet. Forskolin-induced Δ*I*_*sc*_ was observed at all segments and no significant differences were observed among segments (Fig. 4C). These results suggest that high glucose intake may sequentially suppress glucose absorption activity starting in the upper small intestine. It is thought that under these conditions, the same amount of glucose expelled from the stomach is absorbed at a slower rate than under fasting conditions, thus reducing the rapid rise in blood glucose.

### Effect of refeeding on fasting induced increase in SGLT1 transport activity

**Figure 5:**
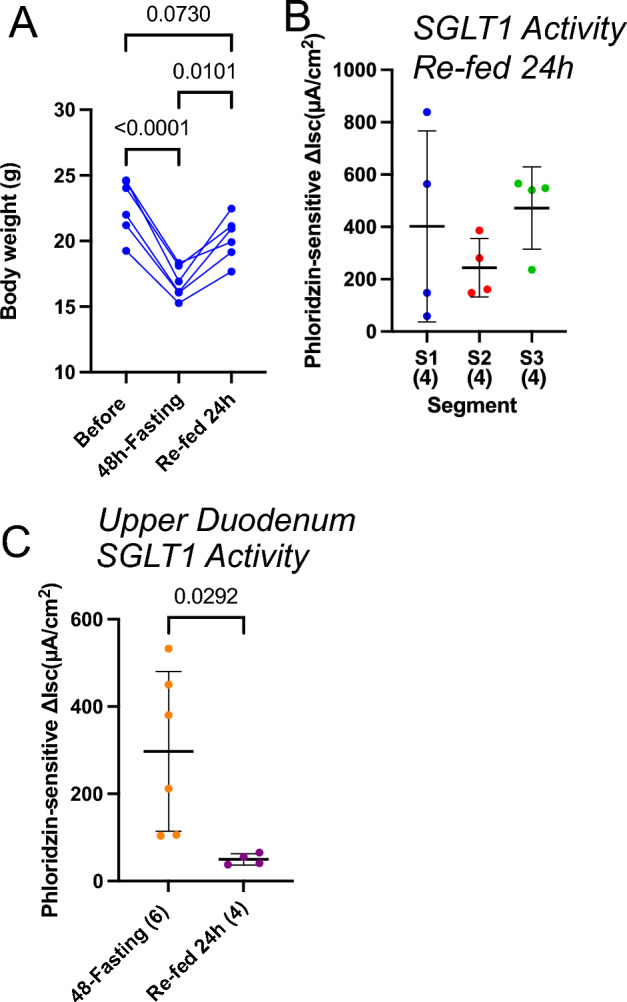
Effect of re-feeding on fasting-induced increase in SGLT1 transport activity. Time course of body weight change after fasting and re-feeding (**A**); results for five animals are shown. The summary of phloridzin-sensitive Δ*I*_*sc*_ (**B**) in S1, S2 and S3 segments 24 h after re-feeding. The summary of phloridzin sensitive Δ*I*_*sc*_ (**C**) in the upper duodenum (proximal to the duodenal papilla) in 48-h fasted mice and 24 h after re-feeding. Data are presented as mean ± SD. Numbers in parentheses indicate the number of animals. *P* values are shown with numbers, One-way ANOVA.

We next examined the time course of inactivation of SGLT1 activity that had been activated by 48 h of fasting. Mice were given ad libitum access to a normal diet (Re-fed for 24 h) after fasting for 48 h. When mice were allowed to re-feed, body weight regain to the levels of pre-fasting was observed (Fig. 5A). Although the variation in phloridzin sensitive Δ*I*_*sc*_ was large in the S1 segments, more robust phloridzin sensitive Δ*I*_*sc*_ was observed upon refeeding than that of the ad libitum fed mice (Figs. 3F, 5B) in the S1 segment. A more detailed study was performed using the upper duodenum, proximal to the duodenal papilla (Fig. 5C). In the refeeding group, SGLT1 activity, which had been observed after 48 h of fasting, was significantly reduced (*P* = 0.0292, *t*-test). These results suggest that the inactivation process begins in the upper small intestine.

### Assessment of non-electrogenic glucose absorption mechanism under fasted conditions

**Figure 6:**
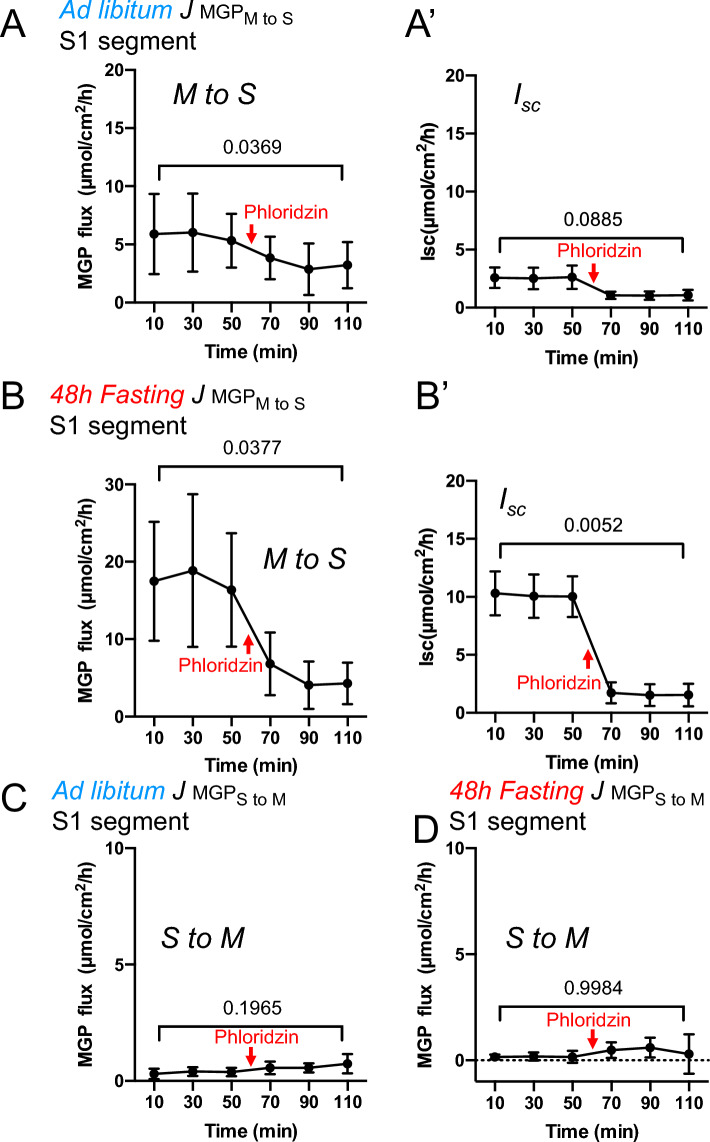
Effect of fasting on SGLT1 and non-electrogenic glucose mediated absorption mechanism in the jejunum. In chambers containing a mucosal preparation from the jejunum (S1), the Ringer’s solution of mucosal (M) side (**A, B**) or serosal (S) side (**C, D**) was replaced with a Ringer’s solution containing 30 mM Methyl α-d-glucopyranoside (nonmetabolizable analog of glucose, MGP) and labeled with ^14^C-MGP at time 0. The unidirectional flux (*J*_M to S_, *J*_S to M_) of MGP was calculated by collecting a sample from the cold side every 20 min. *I*_*sc*_ was measured simultaneously under short circuit conditions (**A′, B′**). Where indicated by arrows, 100 µM phloridzin was added to the mucosal side (at 60 min). Data are the mean ± standard deviation (SD). n = 5. *P* values are shown with numbers, One-way ANOVA.

Kellet has shown that the major mechanism of glucose absorption is via facilitated transporter GLUT2 when the intestine is perfused with high concentrations of glucose^12^. In addition, Wright has also shown that there is a slow absorption mechanism that is not mediated by SGLT1^16^. We could not observe glucose-induced Δ*I*_*sc*_ in the S1 segment in ad libitum-fed mice (Fig. 3A, solid line). These results suggest that a non-electrogenic glucose absorption mechanism may be the major transport pathway in the S1 segment in ad libitum-fed mice. This possibility was investigated by measuring the transepithelial flux of a nonmetabolizable glucose analog, ^14^C-Methyl α-d-glucopyranoside (MGP)^26^. Unidirectional fluxes of MGP and *I*_*sc*_ were measured simultaneously under short circuit conditions in the S1 segment. Since Kellett showed that a high concentration of glucose is required for GLUT2 to insert into the luminal membrane, 30 mM MGP was used in this experiment. We first observed the unidirectional flux of MGP from the mucosal (M) to the serosal (S) side in the S1 segment of ad libitum-fed mice (Fig. 6A). In the absence of mucosal phloridzin, the unidirectional flux of MGP was about 6 µmol/cm^2^/h. Addition of phloridzin resulted in a decrease of the M to S flux by about 3 µmol/cm^2^/h, but the remaining 3 µmol/cm^2^/h was not inhibited. Although it did not reach statistical significance (Fig. 6A′), *I*_*sc*_ was inhibited by phloridzin (∆*I*_*sc*_ = -1.6 µmol/cm^2^/h). The unidirectional flux of MGP from the serosal to the mucosal side (S to M) was about 0.4 µmol/cm^2^/h (Fig. 6C), which was not changed by the addition of phloridzin (Fig. 6C). These results suggest that MGP fluxes in the S1 segment of ad libitum-fed mice are mediated by a transcellular process (3 µmol/cm^2^/h by SGLT1) and a non-electrogenic flux of about 3 µmol/cm^2^/h.

Next, the same experiment was performed using the S1 segment of 48-h fasted mice (Fig. 6B). Under basal conditions, the unidirectional flux of MGP from M to S was about 18 µmol/cm^2^/h. Upon addition of phloridzin, the unidirectional flux was decreased by about 15 µmol/cm^2^/h, but the remaining flux of about 4 µmol/cm^2^/h was not inhibited by phloridzin. The flux of MGP from S to M (Fig. 6D) was about 0.2 µmol/cm^2^/h, which was not affected by fasting or the addition of phloridzin. As shown in Fig. 6B′, MGP-induced Δ*I*_*sc*_ inhibition by phlorizin was about 9 µmol/cm^2^/h.

Together, these results suggest that the major glucose absorption mechanism in the S1 segment under 48-h fasting conditions is mediated by SGLT1. In addition, there is a non-electrogenic absorbable MGP flux of about 4 µmol/cm^2^/h in the S1 segment, which is not affected by fasting.

### Effects of fasting on paracellular ion permeability

**Figure 7:**
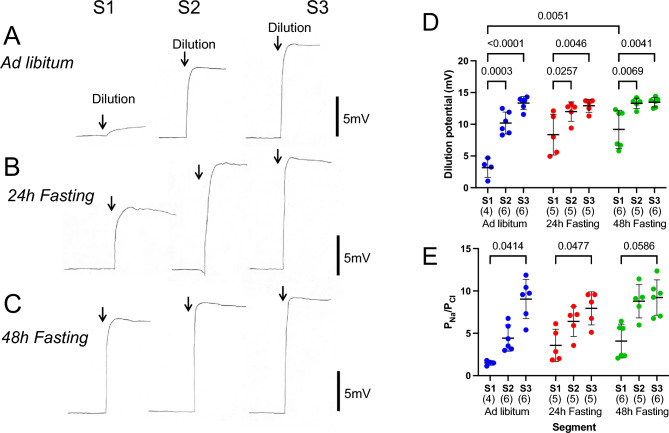
Effects of fasting on paracellular ion permeability. Representative traces of dilution potentials are shown in each condition (**A** Ad libitum-fed mice, **B** 24-h fasted mice and **C** 48-h fasted mice) and each segment (S1: left, S2: middle, and S3: right panel) from six experiments. Where indicated by arrows, mucosal Ringer’s solution (140 mM NaCl) was replaced with Ringer’s solution containing 60 mM NaCl. The dilution potentials were measured under open circuit conditions. The summary of dilution potentials (**D**). The permeability ratio of Na^+^ to Cl^−^, was calculated from the dilution potential using the Goldman–Hodgkin–Katz equation (**E**). Data are presented as mean ± SD. Numbers in parentheses indicate the number of animals. *P* values are shown with numbers, Two-way ANOVA.

The above-mentioned experiments revealed that glucose-induced Δ*I*_*sc*_ (Fig. 3F) and transepithelial electrical conductance were concomitantly increased by fasting in the S1 segments (Fig. 2B). To scrutinize paracellular ion permeability, we measured dilution potential under open-circuit conditions in Ussing chambers^19^. We first measured dilution potential in the *ad-libitum* fed mice (Fig. 7A, D, blue symbols). The NaCl concentration in the mucosal side chamber was diluted from 120 to 60 mM, which resulted in an increment of luminal positive potential. This dilution potential (*ΔPD*) significantly increased from the proximal to the distal segment (*P* = 0.0003 and < 0 0.0001, S1 *vs*. S2 and S1 *vs*. S3, respectively, two-way ANOVA). We next examined the effect of fasting on *ΔPD* (Fig. 7B,C). In the S1 segment, compared to ad libitum-fed mice, larger increases in *ΔPD* were observed in 48-h fasted mice (Fig. 7C,D, green symbols).

P_Na_/P_Cl_, the permeability ratio of Na^+^ to Cl^-^, was calculated from the dilution potential using the Goldman-Hodgkin-Katz equation (Fig. 7E). In ad libitum-fed mice, the median of P_Na_/P_Cl_ was about 2 in the S1 segment, 4 in the S2 segment, and 10 in the S3 segment, indicating that the distal small intestine showed higher cationic selectivity than the proximal small intestine. In 48-h fasting conditions, the median of P_Na_/P_Cl_ in the S1 segment was about 4, suggesting cationic selectivity is increased by fasting in the S1 segment (Fig. 7E, green symbols).

### Effects of fasting on mRNA expression of claudin-2, -7, and -15

**Figure 8:**
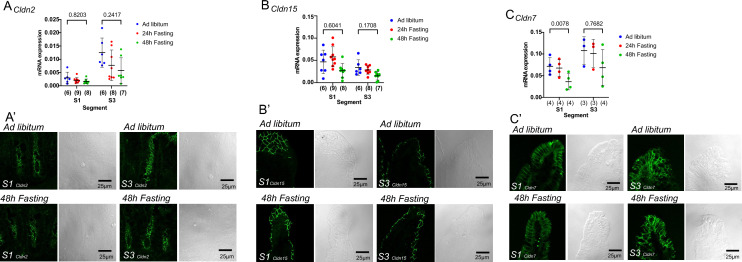
Effects of fasting on mRNA expression of claudins. The effect of fasting on *Cldn2* (**A**), *Cldn15* (**B**) and *Cldn7* (**C**) mRNA expression. Data are normalized to beta-actin. Data are presented as mean ± SD. Numbers in parentheses indicate the number of animals. *P* values are shown with numbers, Two-way ANOVA. Effect of fasting on tissue distribution of claudin-2 (**A′**), claudin-15 (**B′**) and claudin-7 (**C′**). To demonstrate the specificity of the signal, DIC images were obtained at the same time.

A possible cause of permselectivity changes in the paracellular pathways could be altered expression levels of the tight junction claudin proteins. To investigate this possibility, we performed quantitative real-time RT-PCR. We examined the gene expression levels of claudin-2 and -15, which form cation-selective pores in the small intestine^29^. We also examined the changes in gene expression levels of claudin-7, which is considered to be a barrier-forming claudin^13^. Although it did not reach statistical significance, the expression of claudin-2 mRNA was higher in S3 segment than in S1 segment in ad libitum-fed mice (*P* = 0.1692 S1 *vs.* S3, two-way ANOVA, Fig. 8A). With prolonged fasting time, no obvious change was observed. Claudin-15 mRNA expression was not significantly different between S1 and S3 segments in ad libitum-fed mice (*P* = 0.3777, S1 *vs.* S3, two-way ANOVA, Fig. 8B). Claudin-15 mRNA expression was not significantly decreased in 48-h fasted mice in either segment (Fig. 8B). Claudin-7 mRNA expression was not significantly different between S1 and S3 segments in ad libitum-fed mice (*P* = 0.5028, S1 *vs.* S3, two-way ANOVA, Fig. 8C). In the S1 segment, but not in the S3 segment, claudin-7 mRNA level was significantly decreased in 48-h fasted mice (P = 0.0078 and 0.7682, S1 and S3, two-way ANOVA, Fig. 8C). The functional experiments showed that fasting increased glucose-induced Δ*I*_*sc*_, transepithelial electrical conductance (Fig. 2B), and Na^+^ selectivity (Fig. 7D) in the S1 segments. Although an increase in claudin-15, the major cation-selective protein in the small intestine, was expected, there were no distinct changes in fluorescence or structural alterations (Fig. 8B′). Claudin-2, which is also cation-selective, is expressed in the crypts, and no change in fluorescence signal or tissue distribution was observed under fasting conditions (Fig. 8A′). Claudin-7 is mostly expressed in the basolateral membrane, and no significant changes were observed even under fasting conditions (Fig. 8C′).

## Discussion

In the present study, we found that SGLT1 in the upper small intestine is not activated by fasting, but rather is inactivated by feeding. We hypothesized that this could be advantageous for the rapid absorption of small amounts of glucose after fasting, as well as for preventing hyperglycemia during periods of high glucose intake. It should be noted that these results were observed in mice, and further investigation in other species is necessary.

The results of the present study differ from those of previous studies in two significant ways. First, it is generally believed that the absorption activity of sugars increases with increasing intake^27^. The glucose absorption activity in the proximal small intestine in this study showed a pattern of absorption regulation similar to that seen in minerals (Figs. 3F, 4). In other words, when an organism is deficient in minerals, the active transport mechanisms located in the apical membrane of the enterocytes are increased. Conversely, when the organism is not deficient or the intake is high, the total number of transporters in the epithelial cell membrane of the small intestine decreases. Second, immunostaining showed that the protein expression of SGLT1 was higher in the upper small intestine^24^, and if we assume that the SGLT1 expressed in epithelial cells is functional, the results of this experiment are inconsistent with the functional measurements. It is conceivable that SGLT1-mediated glucose absorption in the proximal small intestine under ad libitum fed conditions may not be in operation for some reason. The secretory function induced by forskolin is maintained in the proximal small intestine in ad libitum-fed mice (Fig. 3H), so it is unlikely that the integrity of the intestinal segment is not maintained as it has been shown that CFTR, a Cl^−^ channel responsible for Cl^−^ secretion, is expressed in the villi where SGLT1 is expressed in the upper small intestine, unlike the lower small intestine^30^.

As shown by the lack of phloridzin-sensitive *I*_*sc*_ in Fig. 3F, under fed conditions, SGLT1 on the microvillus membrane may be decreased by the presence of luminal contents and it may become inactive. This explains the decrease in glucose-induced short-circuit currents at the S1 and S2 segments compared to S3, as shown in Figs. 3F and 4B. However, the intestinal epithelium is always regenerating, and a new epithelial covering can be in place by 48 h as the turnover of epithelial cells in the small intestine is 2–3 days^31^. Therefore, after 48 h of fasting, small intestinal function could be considered to be in a default state. In other words, when glucose intake is low, the number of transporters on the apical membrane increases, and glucose is taken up through an active transport mechanism that requires energy. When considering only the upper small intestine, the mechanism for regulating glucose transport activity in this experiment appears to be similar to the mineral regulation mechanism. Alternatively, it can be explained by assuming that the diffusional glucose transport mechanism reported by some groups^8,11^ is the major mechanism in the upper small intestine under ad libitum fed conditions. A (diffusion) transport component not attributable to SGLT1 as measured by the transepithelial flux of MGP of about 3 µmol/cm^2^/h was observed in the upper small intestine, but this component was not affected by fasting (Fig. 6). These results suggest that SGLT1 activity may not be functional in the proximal small intestine in ad libitum-fed mice. As demonstrated by Kellett et al*.*^11,12^, this idea is consistent with research results showing that under conditions of high glucose intake, it is more efficient to take up glucose into the body through diffusion transporters such as GLUT2 than to absorb it through SGLT1, an active transport mechanism that requires energy. We also assessed the involvement of glucose absorption via GLUT2. We obtained a commercial anti-GLUT2 antibody (Proteintech 66,889–1-lg) and used it in this experiment. However, the antibody did not work in native tissues, either by western blot or immunohistochemistry. Nevertheless, it worked in MDCK cells expressing mouse GLUT2, and GLUT2 was found in the apical membrane (data not shown), consistent with previous studies^32^. These results indicate that future experiments are needed to define the role of GLUT2 in apical glucose transport in the intestine.

In this study, the highest absorption capacity in the upper small intestine was observed during fasting, whereas little glucose absorption was observed during a 3-day high glucose diet or during free feeding. Glucose absorption activity was observed in the ileum regardless of the feeding conditions. As an interpretation of these findings, there is a possibility that the intestinal absorption mechanism is automatically regulated based on the glucose concentration in the lumen of the small intestine, since in nature the amount of glucose intake in the next meal cannot be predicted. This explains why only SGLT1 and not SGLT2 (as in the kidney) is expressed in the small intestine; it protects against hyperglycemia by absorbing too much glucose too quickly.

What then is the physiological significance of the increased and decreased functional activity of SGLT1 in the proximal small intestine? As systems that efficiently absorb solutes from the lumen in tubular structures, the longitudinal expression of transporters in the gastrointestinal tract and the renal tubules are similar. For example, in the case of Na^+^ (re)absorption, in the proximal part of the intestinal and renal segments, Na^+^/H^+^ exchangers are expressed, and constitutive absorption occurs, while in the distal part, the expression of epithelial Na^+^ channels, which are Na^+^ scavengers, is regulated in response to biological conditions. However, with regard to the mechanism of glucose absorption, the distribution of glucose transporters on the longitudinal axis and their characteristics are different in the kidney and the gastrointestinal tract, but the physiological significance of this difference has not been explained^33,34^.

When comparing the long-axis distribution of glucose transporters in the kidney tubules and the gastrointestinal tract, the proximal portion of the tubule expresses the high-capacity type SGLT2, while the distal portion expresses the low-capacity, high-affinity type SGLT1, which efficiently reabsorbs glucose in the distal portion. It is not clear why a low-capacity, high-affinity type of SGLT1 is expressed in the proximal segment of the small intestine and the reason SGLT2 is not expressed in the small intestine is not explained. One explanation for the difference in the expression sites of SGLT1 in the kidney and the digestive tract is that the glucose concentration expected to be loaded in the proximal tubule can account for this phenomenon. In the case of the kidney, the blood glucose level in healthy humans is maintained at around 5 mM, and the concentration in the glomerular filtrate only changes within an expected range. However, in the case of the digestive tract, the glucose concentration during meals varies depending on the meal, and it is impossible to predict the glucose concentration in the next meal in nature. This long-axis distribution of SGLT1 in the small intestine is suitable for rapid uptake of small amounts of glucose. However, the presence of SGLT1 in the proximal segment could be considered disadvantageous from the perspective of homeostasis, as it leads to the uptake of large amounts of glucose into the body when consuming high levels of glucose, resulting in hyperglycemia. It is reasonable to assume that the small intestine autoregulates its ability to absorb glucose to prevent hyperglycemia when large amounts of glucose are ingested. Although the mechanism of this regulation could not be clarified in this study, one possibility is that SGLT1 is degraded by digestive enzymes in the lumen. Interestingly, in rats, it has been shown that SGLT1 in the upper small intestine is degraded by the contents of the lumen^35^. As for the observed autoregulation function in the upper small intestine of mice, further research is needed, including in other species.

During a 3-day high glucose diet, the glucose absorption capacity decreased in S1 and more so in S2 (Fig. 4A). Furthermore, after 24 h of refeeding following a 48-h fast, the glucose absorption capacity was further reduced in the upper duodenum compared to S1 (Fig. 5C). This indicates that the glucose absorption activity sequentially decreases from the upper small intestine depending on the amount of glucose in the lumen. Physiologically, this suggests that during high glucose absorption, glucose is slowly absorbed in the middle and lower small intestine. Supporting this hypothesis, regardless of the diet conditions, glucose is continuously absorbed in the ileum (Figs. 3F, 4B, 5B), which is consistent with the absence of glucose in the ileal contents. This mechanism suggests that the intestinal tract can automatically adapt to unexpected glucose intake in nature. Further research is needed to elucidate the system that senses glucose in the lumen.

Fasting increased the transepithelial conductance, an index of ionic permeability between epithelial cells, that was observed in the upper small intestine (Fig. 2B). Examination of the ion permeability of the paracellular pathway revealed that the increase in conductance was due to an increase in Na^+^ permeability (Fig. 7). The increase in cation selectivity in the tight junctions induced by fasting may be important for supplying the lumen with the Na^+^ necessary for glucose absorption. Claudin-15 has been shown to play an important role in cation selectivity in the epithelium of the small intestine^17,29^. However, in the present study, there was no obvious change of mRNA expression observed (Fig. 8B). Decreased expression of claudin-7, which has a barrier-like function, was observed (Fig. 8C). Further studies are needed to elucidate the mechanism of cation selectivity in tight junctions, which is increased by fasting.
